# Lightweight On-Device Detection of Android Malware Based on the Koodous Platform and Machine Learning

**DOI:** 10.3390/s22176562

**Published:** 2022-08-31

**Authors:** Mateusz Krzysztoń, Bartosz Bok, Marcin Lew, Andrzej Sikora

**Affiliations:** NASK PIB, Kolska 12, 01-045 Warsaw, Poland

**Keywords:** malware detection, Android security, Koodous platform, models aging, machine learning, neural networks, lightweight models, edge computing

## Abstract

Currently, Android is the most popular operating system among mobile devices. However, as the number of devices with the Android operating system increases, so does the danger of using them. This is especially important as smartphones increasingly authenticate critical activities(e-banking, e-identity). BotSense Mobile is a tool already integrated with some critical applications (e-banking, e-identity) to increase user safety. In this paper, we focus on the novel functionality of BotSense Mobile: the detection of malware applications on a user device. In addition to the standard blacklist approach, we propose a machine learning-based model for unknown malicious application detection. The lightweight neural network model is deployed on an edge device to avoid sending sensitive user data outside the device. For the same reason, manifest-related features can be used by the detector only. We present a comprehensive empirical analysis of malware detection conducted on recent data (May–June, 2022) from the Koodous platform, which is a collaborative platform where over 70 million Android applications were collected. The research highlighted the problem of machine learning model aging. We evaluated the lightweight model on recent Koodous data and obtained $f_{1}=0.77$ and high precision (0.9).

## 1. Introduction

With more than 2.5 billion active users (75% of the global market), Android will become the world’s most popular operating system in 2022 [1]. It is closely related to the development of the broad mobile technology market. Android is installed on all available mobile devices, including smartphones, tablets and netbooks. Applications are distributed through the Google Play Store, which has over 2.5 million of them [2]. However, as the number of devices with the Android operating system increases, so does the danger of using them. Many applications may contain malware, so it is important to protect devices against it. Security is essential when smartphones are increasingly used to authenticate critical activities (e-banking, e-identity).

At the same time, Android malware detection systems are constantly evolving. Facing security challenges in recent years, several anti-malware tools have been developed for Android, among which the following deserve special mention Norton (https://norton.com, accessed on 20 July 2022), McAfee (https://mcafee.com, accessed on 20 July 2022) or Virustotal (https://www.virustotal.com, accessed on 20 July 2022) tool. The last one is particularly noteworthy as it aggregates many engines for detecting malware applications. However, despite a wide range of tools, a significant number of devices remain unprotected (e.g.,, in the US, only half of mobile devices have an antivirus installed [3]).

The increasing role of mobile devices and their lack of safety have incited app developers to take more responsibility for the protection of the user device. BotSense Mobile system was created to fill that need. The system is distributed as an SDK library integrated into the client’s mobile application. Currently, it is implemented in the mobile apps of five Polish banks and institutions, reaching approximately three million users.

In this paper, we concentrate on the novel functionality of BotSense Mobile: the detection of malware applications on a user device. We propose an integration of a commercial system for creating whitelists and blacklists. However, the application appearance in the external system is subjected to some latency. Thus, we propose to apply machine learning (ML) to detect unknown malicious applications. However, the ML-based models that classify Android applications as malicious or safe may degrade over time [4]. For example, the article [5] published by the well-known producer of antivirus software Kaspersky presents that the degradation of an ML malware detector results by 20% in just three months. The problem of aging was addressed in works [4,6]. However, the proposed methods slow down model aging only in the case of ML models based on API call analysis.

Based on the overview of previous ML-based works, we identified a lack of auto-updating tool for malware detection that satisfies the BotSense Mobile requirements, addresses the problem of model aging, and uses the ever-growing database as the source of knowledge. Thus, this paper extends the BotSense Mobile malware detection module with a self-developed lightweight, on-device ML-based tool to detect unknown malicious applications. The main contribution of the paper are:

- Comparison of Android malware data sources as a potential source of knowledge for on-device, auto-updating commercial solutions;
- A description of Koodous platform data characteristics—to the best of our knowledge, this is the first paper treating so comprehensively on Koodous malware database;
- A description of the on-device malware detection system using a blacklist and whitelist methods supported by a lightweight neural network model;
- Comprehensive empirical analysis of malware detection with machine learning (ML), conducted on recent data (May–June, 2022) from the Koodous platform, with a special focus on the problem of machine learning model aging.

The rest of the paper is organized as follows. First, in Section 2, we introduce the BotSense Mobile system and architecture of the module responsible for malicious app detection. Primarily, we indicate the ML-based module’s limitations resulting from the BotSense Mobile architecture. In a Section 3, we present an overview of commercial malware databases, with particular emphasis on the one chosen as the primary source of data (the Koodous platform). Then, in Section 4, we provide an overview of existing ML-based systems for Android malware detection. Then, we described our tool for unknown malware detection and the level of malignancy assessment. Section 5 presents the results of the comprehensive empirical analysis of the developed tool with a particular focus on the problem of aging ML models. Finally, we conclude our work in a Section 6.

## 2. BotSense Mobile

BotSense Mobile is a tool developed by NASK in 2018 as an extension of a successful BotSense system (https://en.botsense.pl/, accessed on 20 July 2022) for securing banking sites (currently securing 16 million Internet banking accounts). The tool is designed for phones with the Android operating system. It is distributed as an SDK library that needs to be integrated into the client’s mobile application (e.g., e-banking applications). The main functionalities of the application are:

- Verification of device integrity;
- Verification of the integrity of the client’s protected application;
- Verification of the network settings;
- Providing the client with a fingerprint of the device and browser.

Current development is concentrated on the behavioral profiling of application users, detecting anomalies in the operation of a protected client application and detecting malicious applications installed on the phone using ML. This article presents the preliminary results of the research in the last area.

### 2.1. Architecture

**Figure 1:**
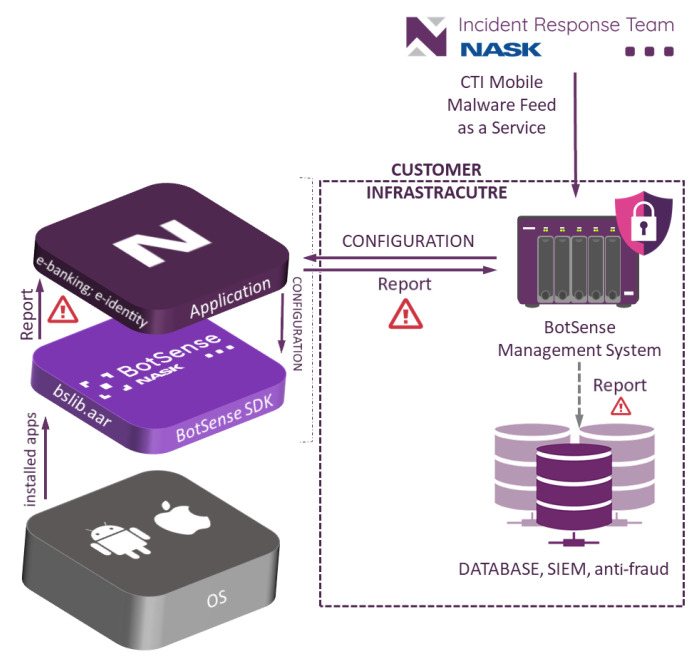
BotSense mobile deployment scheme.

BotSense Mobile deployment scheme is presented in Figure 1. BotSense Mobile itself is composed of two components:

- BotSense Management System—component is installed within the client infrastructure. It is responsible for updating the configuration of the BotSense SDK (e.g., new blacklist/whitelist, new ML model) and consuming and propagating alerts raised by BotSense SDK. This component is fed with data by NASK systems;
- BotSense SDK—component is installed within the user device with the client’s application. This performs all security analyses to avoid sending sensitive user data outside the device.

The client application serves as a proxy between BotSenseSDK and BotSense Management System. The system sends alerts to the client’s systems (e.g., SIEM, antifraud). Based on those alerts, the client’s system can perform further steps to mitigate the risk, e.g., block the banking application or demand additional authorization.

### 2.2. Malware Detection Module

**Figure 2:**
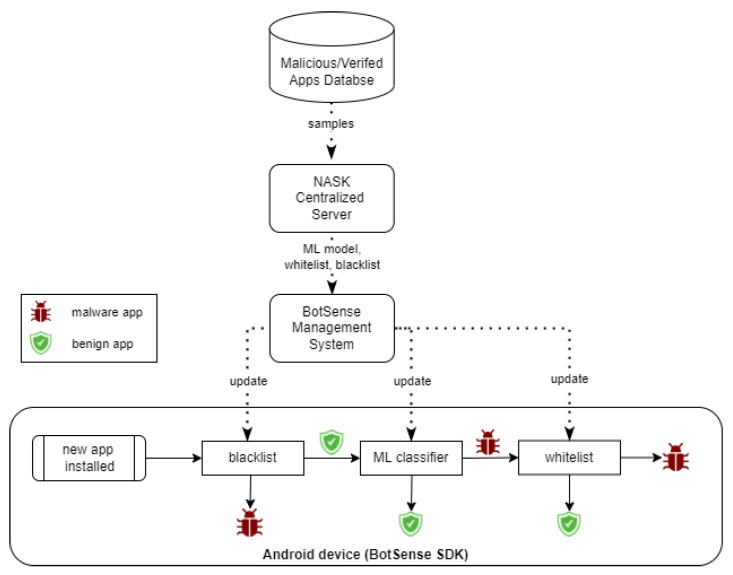
Malware detection module architecture.

A malware detection module analyzes each application installed by the user on the device. The module comprises three sub-modules: whitelist classifier, blacklist classifier and an ML classifier Figure 2). Blacklist and whitelist classifiers are fed by a centralized service based on an external data source with known malicious and verified applications, accordingly. The role of the third sub-module, the ML classifier, is the detection of suspicious applications that have not yet been added to the external database. The ML model is rebuilt periodically by the centralized service. The BotSense Management System is deployed within client infrastructure and serves as a proxy between NASK services and BotSense SDK.

The evaluation of a new application is sequential: first, the blacklist classifier verifies the application. If the application does not exist on the blacklist, then the ML classifier checks whether the application is similar to the known malware. If not, the application is recognized as safe. Otherwise, the application is identified as malicious. Finally, the application is checked against the whitelist to reduce the false positive rate.

The decision to perform all analyses within the user device significantly influences the malware detection module—to assess application maliciousness, only data extractable within the device are available for analysis. Thus, approaches as API calls analyses [7,8], static code analyses [9,10] or dynamic code analyses [10] are not possible.

## 3. Malware Data Sources

Several sources of information about the maliciousness of the known applications exist on the market. The ideal source should contain an extensive and ever-expanding collection of applications. With each application there needs to be an associated label indicating whether the application is malware. Additionally, some applications can be marked as verified benign applications. Those applications can be used to create whitelists. Of course, the quality of labeling is crucial as well. However, it is a difficult task to obtain high-quality labeling with a large number of analyzed samples at the same time. In terms of BotSense integration, the data source should be easy to use from the programming level (provide an API) and allow large amounts of data to be collected in a reasonable amount of time.

### 3.1. Comparison of Existing Sources

We identified several Android malware data sources, both free and commercial. In the scientific literature, the most popular choices for conducting research are Drebin [11,12,13], AndroZoo [13,14], and VirusShare [15,16]. Drebin is a static dataset containing samples from August 2010 to October 2012 only. On the other side, the Androzoo dataset is the biggest state-of-the-art dataset among the open ones. However, API is no longer available (https://androzoo.uni.lu/access, accessed on 20 July 2022). Additionally, data cannot be used commercially. Applications in Androzoo do not have an associated app release date [17], making research on aging models impossible. VirusShare is the general malware data source, also containing numerous Android applications. However, the use is restricted to selected institutions only, and no API is available (year-round packages are available for download).

Contrary to the above solutions, ever-growing big datasets of Android applications, both malicious and benign, with easy access via API, are available on Koodous (*Koodous Documentation*, https://docs.koodous.com, accessed on 20 July 2022) and Virustotal (https://virustotal.com, accessed on 20 July 2022). Koodous is a platform that has already collected over 70 million Android applications. Each application is available to download with both static and dynamic analysis performed with external tools (Cuckoo Sandbox (https://cuckoosandbox.org/, accessed on 20 July 2022), Androguard (https://github.com/androguard/androguard, accessed on 20 July 2022) and DroidBox (https://github.com/pjlantz/droidbox, accessed on 20 July 2022). The research community performs labelling applications based on the analysis results mentioned above. All data are available via REST API, with usage limits dependent on pricing plans. To our knowledge, the list of applications available in Virustotal and Koodous are similar, and both solutions are related. However, the advantage of the Koodous platform is the possibility to upload application samples to be analyzed by the community. This feature is highly important as the NASK National Response Team (https://www.nask.pl/pl/dzialalnosc/nauka-i-biznes/nask-incident-response/3985,NASK-Incident-Response-Team.html, accessed on 20 July 2022) monitors the local market to identify applications, especially dangerous for Polish users. Thus, in the rest of the paper, we present the results of the preliminary examination of the Koodous platform (commercial version) as the main data source for BotSense Mobile.

Apart from in Android-specific data sources, the Android malware samples can be found in data sources of any malware: MalwareBazaar (https://bazaar.abuse.ch, accessed on 20 July 2022), Joe Sandbox Cloud (https://www.joesandbox.com/, accessed on 20 July 2022). However, Android samples in those databases are infrequent. Finally, there are two relatively new sources: APKLAB.IO (https://www.apklab.io, accessed on 20 July 2022) (available beta version with no API, open for researchers only) and APKDETECT (https://app.apkdetect.com, accessed on 20 July 2022) (still restricted to selected researchers) that could be considered in the future.

### 3.2. Koodous Platform Data Characteristic

There are only a few articles presenting the study of classification models for dangerous applications based on data from the Koodous platform [18,19]. In [19], research on malware family prediction was presented, although the most recently used data are relatively old (May 2016). In [18], no information about data characteristics (beyond the size of data) was provided.

In the Koodous platform, applications are searchable using a hash function from the APK file (e.g., SHA256). The description of each application includes, i.a., name, the date the application was added to the database and the results of the static analysis (including Android Manifest) and, optionally, the dynamic analysis. Additional information associated with the application is *ranking* with a default value 0. Since Koodous is a collaborative platform, the rating value is based on analyst votes, where each vote increases or decreases the rating by 1.

To evaluate the volatility of the ranking value, we performed the following experiment: on 21 June 2022, we downloaded 90,000 applications added from 3 June to 21 June. Applications were distributed equally in this period. Three weeks later, we downloaded rating values for those applications again and compared the new rating value with the old one. Of all the samples, only 0.341% (307 applications) changed their rating. No correlation was observed between the number of applications with a changed rating and the application age. Thus, it can be assumed that the vast majority of analysts automatize the application evaluation. Moreover, in all cases, the rating value changed from 0 to −2, which can be interpreted as a change from potentially benign to detected malware. This might result from detecting new malware families by society and marking related applications from the past.

**Figure 3:**
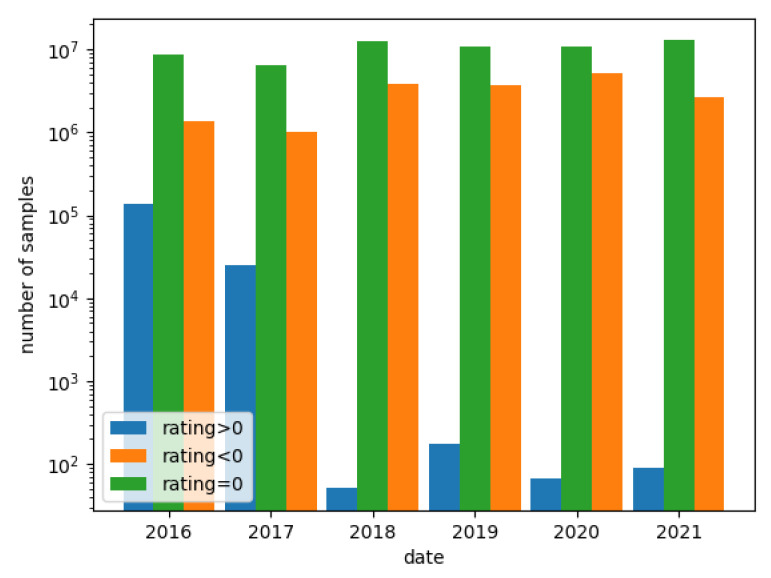
Data distribution in the Koodous platform from 2016 to 2021 (note a logarithmic scale).

Figure 3 presents the distribution of applications during the period 2016–2021, which were categorized by a sign of rating.

As shown in Figure 3, in the year 2018, the number of benign applications (rated above 0) decreased drastically and the number of malicious applications (rated below 0) increased. However, applications with a rating=0 remain roughly the same all the time.

Due to the decrease in the number of benign applications on the Koodous platform over time, it was decided to relax the criterion of considering the application as benign—it was assumed that the scanned applications with a score of 0 are benign.

### 3.3. Koodous Platform as BotSense Datasource

BotSense mobile was integrated with the Koodous Platform via REST API. Each hour, new analyses of applications are downloaded from the feed as one package. The community uses those analyses to rate the applications. Due to the low rating volatility, we download each new application’s ranking the very next day. The blacklist is supplemented with applications with the detected flag (rating<−1), and the whitelist with applications contains a positive rating (rating>0). Additionally, the rating of old applications with a rating equal to 0 might be periodically polled. Unfortunately, this operation cannot be efficiently implemented due to a lack of vote feed in the Koodous API.

In the case of ML classifier, due to the rarity of samples with a positive rating, applications with a rating≥0 are assumed to be benign (in fact, within those applications, there might be rare non-identified malware examples, e.g., those representing a new malware family). Samples with rating<−1 are labeled as malware. Such assignment of labels affects the aim of the classifier—its purpose is to detect new malicious applications similar to those marked as malicious by the Koodous community before they are uploaded and rated in the Koodous platform. However, those new malware families can be only detected if they are similar to the old ones.

## 4. Machine Learning for Unknown Malware Detection

Due to a large amount of malware on Android devices, malware detection has been a significant subject of research among the ML community. In 2021, the systematic review [20] of the methods based on ML was published. Those methods can be divided into three groups according to the type of analysis used to gather data about the application: static analysis, dynamic analysis and hybrid analysis. Static analysis is checking the application before letting it run on the device—the subject of analysis is the application’s source code, including configuration files and metadata. Within the static analysis, the manifest-based analysis can be distinguished. Dynamic analysis checks the behavior of the application during runtime only. The hybrid analysis combines these two approaches, thus having the most data on which to build a detection model.

Due to the architecture of the BotSense system, we are limited to the manifest-based analysis. In this section, we provide an overview of recent works that use manifest-related information to detect malware applications. Then, we verify whether the ML techniques can be used to build a lightweight, high-quality model based on Koodous data to detect those malicious applications that were not yet added to the Koodous platform but have already been downloaded by the user.

Since amount of data in Koodous grows quickly, the created model may become outdated just as fast. This section presents preliminary research results using data from a two-month period. The main focus is the problem of model aging and the methodology for its automatic refreshing.

### 4.1. State of the Art

Malware detection based on manifest-related features has been the subject of many recent research works. The most important papers are shown in Table 1.

Fauzia Idrees et al. proposed malware detection using several machine learning classifiers [21]. They collected Android applications from numerous datasets, achieving an accuracy of 99.8%. In [22], random forest-based model was trained and tested on the OmniDroid dataset. OmniDroid was created partially based on the applications provided by the Koodous Platform but contains data from the 2018 year only. Coronado-De-Alba et al. [23] presented the Random Forest model as well, and focused on comparing various feature selection strategies; they concluded that feature selection has no significant impact on the classification quality. However, Kouliaridis et al. showed that by, using dimensionality reduction techniques, PCA and t-SNE—the scores of eight well-known classifiers—increased [24]. Selecting relevant features was the objective of this paper [25] as well. The findings of this work are applied in our approach. Noteworthy, the authors differentiate unknown and known malware detection tasks and show decay in models’ effectiveness for newer applications. The feature selection was the objective of the paper [26] as well.

An interesting approach was presented in [27] where ensemble models combined of logistic regression, MLP, and SGD models are presented. Whereas, in [28], the authors used four KNN-based models to detect malware, they selected the 300 most important static features using a random forest regressor. This allowed achieving high accuracy results between 90% and 99%.

The aforementioned papers have some limitations according to the objectives presented in this work. First, in works [22,23,28], static analysis takes into account not only the manifest file but also API calls information. This information is not extractable within mobile devices due to Android safety mechanisms. Secondly, works [21,22,23,25,26,27] did not consider the date of application creation when splitting a dataset into training and testing datasets. Thus, the presented accuracy of the classifiers relates to the detection of known malware families, which is a significantly easier task. Consequently, the problem of model aging is not considered as well. Another issue relates to the requirement to transfer and run the classifier on the edge device. As we point out in Section 4.3, the most commercially mature solutions concern neural networks, which are used only in works [21,24,26,27,29]. Finally, all works were carried out on static, non-growing sets, which means that it is impossible to update models with the emergence of new malware families. One exception is works that only use the ever-expanding, up-to-date source of applications (e.g., Google Play Store or APKPure) in conjunction with some malware detection engine (e.g., VirusTotal) [21,23,29]. However, a strategy for updating the model over time has not been proposed and examined. Additionally, such an approach’s effectiveness would strictly depend on the quality of the applied malware detection engine. In this paper, the Koodous Platform is used as both a source of applications and a detection engine (community voting).

The work [29] is the most significant one from the perspective of our research. In this paper, several machine learning models were created using manifest-based features only. The client–server architecture is provided. However, the edge device sends data about a newly installed application to the server. Due to user privacy, such data transfer is impossible within the BotSense system. The advantage of the work is that the test dataset was composed of applications significantly younger than those in the training dataset (at least in the case of the malicious ones). However, the final datasets were composed of applications from various sources. The methodology for selecting applications for the dataset, especially from Google Play Store, is unclear. The age distribution of data is also not provided. Our doubts are raised by the fact that, with a heterogeneous data source and/or with possible wrong sampling methodology, malicious applications could be easily distinguished from benign samples because of hidden correlation (e.g., if benign applications are significantly younger than malicious ones or if benign and malicious applications belong to different theme categories, while categories may differ in the prevalence of malicious applications). As a result, the machine learning method would create a model that discriminates well another feature of data (e.g., the age of the application), which, due to the structure of the set, strongly correlates with the maliciousness of the application. In our work, a homogeneous data source is considered.

### 4.2. Feature Extraction

Each sample is described with a features vector to identify potentially malicious applications. Due to the evaluation of the application on the device, the list of possible functions is limited to the data that can be extracted on the Android phone. Nevertheless, numerous features can be extracted on the mobile device, so paying attention only to those helpful in classifying them as benign or malicious is essential. However, due to the dynamic character of the problem, the list of features should not be excessively restricted, as features that do not correlate with the maliciousness of the application in case of recent data can correlate for future malware families. On the other hand, an excessively large set of features can make the task more difficult.

One source of features is a list of permissions that the application requires to provide the user with all functionalities. Most malware requires some specific permissions to perform an attack. However, there are over 200 permissions defined in Android now, most of which are benign (e.g., the one that allows an application to find out the space used by any package). In [25], only 22 permissions were selected as relevant in the task of malware detection. Additionally, the Android itself defines some permissions as dangerous, as they give access to restricted data and actions that can significantly impact the system and other applications [30]. Finally, we extended the list of permissions with those identified as informative in another research conducted on the Koodous database [31]. The final list of considered permissions is presented in the Appendix A. Each permission corresponds to one binary feature indicating whether the application requires the given permission. Additionally, a feature equal to the number of those permissions present in the application was defined.

Apart from permission-related features, both the Koodous platform and on-device analytical tool allow access to other features resulting from the Manifest file. The ones that may have an impact on the maliciousness of a given application are:

- The number of screen names of the mobile application;
- Whether the Apache Cordova framework was used when creating the application;
- Whether the application is signed with a certificate;
- Whether filters are used;
- The maximum number of API supported Android versions;
- The number of used dangerous filters (listed in Appendix A);
- Whether there are any user-defined permissions;
- Whether the list of application classes with specific functions is non-empty (providers);
- Whether the list of application classes with specific functions is non-empty (receivers);
- Whether the list of application classes with specific functions is non-empty (services);
- The number of url addresses that the application uses;
- Whether the name of the signature file is different from the default (“META-INF/CERT.RSA”).

Finally, each application is described with 71 features.

### 4.3. Methods

The model is built in the central server, where data from Koodous are collected. However, due to the requirement that no data about the application can be transferred outside the device, the created classification model needs to be transferable and executable on the device. The list of ML libraries that support building, serializing and finally executing classifiers on the mobile device is limited:

- **m2cgen** (https://github.com/BayesWitnesses/m2cgen, accessed on 20 July 2022)—the library that allows to convert some learning models to the appropriate native JAVA code. Unfortunately, the number of machine learning methods compatible with the library is limited. Compatible methods include those dominated by linear and tree approaches (including ensemble models). Sending models as pure JAVA code is not recommended. Additionally, the library does not yet have the first major version. However, it is still under development and can be an option in the future;
- **sklearn-porter** (https://github.com/nok/sklearn-porter, accessed on 20 July 2022)—the library dedicated to converting models created using the machine learning library sklearn. The result of the conversion is the JAVA code representing the decision tree. As with m2cgen, none is the first major version of the library. Additionally, the last changes to the library are dated for 2019;
- **PyTorch** (https://pytorch.org, accessed on 20 July 2022)—the machine learning framework for building models based on neural networks (NN). It has a built-in conversion option for any neural network to the TorchScript. The script can be imported for a device with the Android operating system using a dedicated library that is also part of PyTorch. The framework has the first major version constantly developed;
- **TensorFlow Lite** (https://www.tensorflow.org/lite, accessed on 20 July 2022)—a framework similar to PyTorch. The main difference is the lack of support for some elements of neural networks within TF Lite.

Due to the maturity of the solutions, the PyTorch framework was chosen for the development within BotSense Mobile. Choosing PyTorch over TensorFlow is due to the wider compatibility with mobile devices. However, training neural network models with high accuracy requires significant computational effort due to the need for proper neural architecture search and hyperparameter optimization. Thus, we use gradient boosting classifier (GBC) in the first research stage for discovering knowledge in data and learning the applications’ characteristics without implementing a complex model. GBC is one of tree-based ML algorithms. We chose the tree-based algorithm as the reference method as this group of algorithms is commonly used and achieves good efficiency in state-of-the-art works.

#### 4.3.1. Neural Network Model

The PyTorch library enables the easy implementation of the neural network model. The aim of the model is to assign a binary label to the input feature vector. The model we investigate consists of one input layer, three hidden layers and one output layer. The input layer comprises as many neurons as the number of features describing an application, i.e., 71.

Android application samples are fed to the input layer. The values of input neurons are multiplied by appropriate weights and summed up in the first hidden layer. Multiplying the values by weights and summing them up in the next layer is also performed in each hidden layer. Then, each neuron in hidden layers is passed through the ReLu ($\varphi \left(x\right)=x^{+}=max\left(0,x\right)$) activation function. Additionally, there is a dropout layer (with the same dropout probability for each) after each hidden layer. The dropout layer randomly removes some neurons from the hidden layer. It has been proven that it can improve the quality of network performance [32].

At the last layer, there is a single neuron that returns a value with no limitation. To obtain a value in the range [0,1], the output value of this neuron is passed through a sigmoid activation function:

$$y^{*}=\frac{1}{1+e^{-y}}$$

where *y* is the output value of the neuron in the last layer and $y^{*}$ is the model output value. The value in range [0,1] can be easily transformed into binary label: 1 (malicious) if model output value $y^{*}$ is greater than 0.5 and 0 (benign) otherwise.

**Figure 4:**
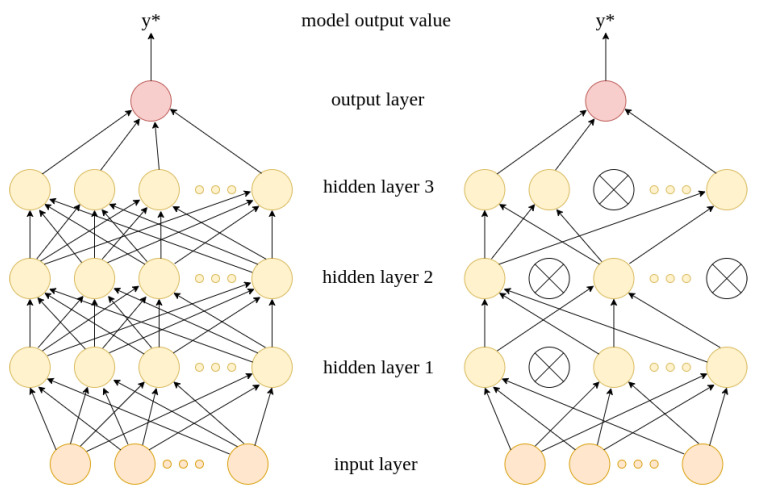
Neural network structure (**left**) with and (**right**) without dropout layer.

The neural network structure is shown in Figure 4.

In the learning process, the training samples (feature vectors) are given on the input layer and appropriate labels on the output. Then, the values of weights $w_{kj}$ between *k*-th and *j*-th neurons in adjacent layers are optimized. The output of each neuron is defined as:

$$o_{j}=\varphi \left(net_{j}\right)=\varphi \left(\sum_{k=1}^{n}w_{kj}o_{k}\right)$$

where $o_{j}$ is output of *j*-th layer, φ is the ReLu activation function, $net_{j}$ is the input of the *j*-th neuron, and $w_{kj}$ is weight between *k*-th and *j*-th neurons.

The model output error *E* is weight-optimized using the backpropagation approach:

$$\frac{\partial E}{\partial w_{ij}}=\frac{\partial E}{\partial o_{j}}\frac{\partial o_{j}}{\partial w_{ij}}=\frac{\partial E}{\partial o_{j}}\frac{\partial o_{j}}{\partial net_{j}}\frac{\partial net_{j}}{\partial w_{ij}}$$

Thanks to the backpropagation algorithm, the model updates its weights in subsequent iterations. After each iteration, the model output error is minimized. The trained model can assign labels, in our case, as malicious or benign, to the given sample.

#### 4.3.2. Gradient Boosting Model

Once a sample is classified as malicious, it is also necessary to calculate the approximate degree of its malignancy. For this purpose, a supervised ML model can be used to predict this degree, taking the rating of the samples as a label. Note that the labels are in total order, so we decided to use a regressor to predict the degree of malignancy, using the float value on the output. We chose the gradient boosting regressor (GBR) from the sklearn library. It is one of the machine learning models whose task is to minimize the loss function using the gradient method—similar to a neural network. A unique approach to solving this problem, in the case of this model, is the creation of increasingly newer decision trees, where each subsequent one minimizes the loss function. The trees create the so-called random forest.

### 4.4. Metrics

An appropriate metric must be chosen to check the quality of the learned model or to compare several models.

According to the current and predicted label, the classification result is assigned to one of the following categories:

- **TP (True Positive)** —both the actual and the predicted label are positive;
- **FP (False Positive)**—the actual label is negative and the predicted label is positive;
- **FN (False Negative)**—the actual label is positive and the predicted label is negative;
- **TN (True Negative)**—both the actual and the predicted label are negative.

In case of problems where relevant and non-relevant elements can be distinguished (e.g., malware and benign applications, accordingly), metrics such as precision and recall are more informative than accuracy.

A high level of *precision* informs about a low number of false positives among all samples predicted as positive:

$$precision=\frac{TP}{TP+FP}$$

In the case of malware detection, a high precision value indicates that most of the applications labeled as malware are indeed malicious.

On the other hand, detecting as much real malware as possible (little FN) is also important. The measure that informs about results completeness is *recall*:

$$recall=\frac{TP}{TP+FN}$$

The combination of the information provided by precision and recall is metric $f_{\beta }$:

$$f_{\beta }=\left(1+\beta^{2}\right)\frac{precision\cdot recall}{(\beta^{2}precision)+recall}$$

Parameter β is used to assign precision and recall appropriate weights, depending on the preferences in the given use case. In this research, sensitivity and precision are given equal weight. Therefore, β = 1 was chosen.

This paper examines the variability of the $f_{1}$ measure over time. Due to the variant percentage of malicious applications among all applications on consecutive days, we decided to balance data with the oversampling strategy each day, so that the results are comparable.

In the case of regression, several metrics can be used to analyze the results. One of the most frequently used ones is root mean square error (RMSE). The value of this measure is equal to the quadratic mean of differences between actual and predicted values:

$$RMSE=\sqrt{\frac{\sum_{i=1}^{N}{\left(\hat{y_{i}}-y_{i}\right)}^{2}}{N}}$$

where:

- $y_{i}$—the prediction for the sample $x_{i}$;
- $\hat{y_{i}}$—the actual value for the sample $x_{i}$;
- *N*—the number of predicted samples.

Another metric is the mean absolute error (MAE). This metric value is more intuitive as it is the average of all absolute errors:

$$MAE=\frac{\sum_{i=1}^{N}\left|\hat{y_{i}}-y_{i}\right|}{N}$$

This metric answers how much the predicted value differs from the actual one on average. The lower the value of RMSE and MAE, the better the regression is.

### 4.5. Automated Model Generation

**Figure 5:**
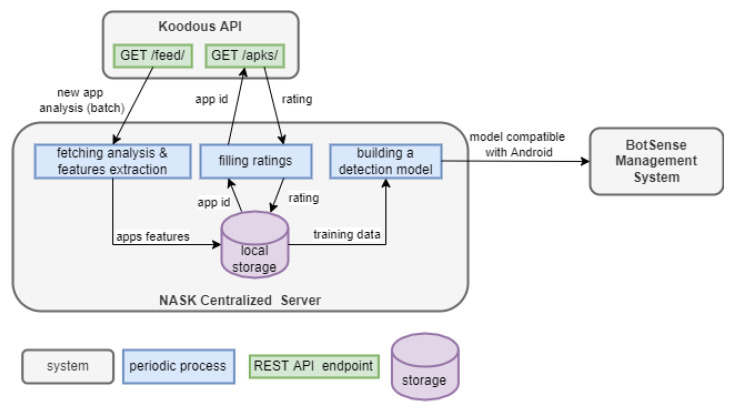
The architecture of the automated model generation mechanism.

Due to malware’s ever-changing characteristics, the model must be constantly retrained with the latest applications from the Koodous platform. Thus, the automatic mechanism of new data collection and new model generation was implemented (Figure 5). The mechanism is composed of three periodic processes:

- Fetching analysis and features extraction (run once an hour)—the process fetches one batch of all applications analysis generated by the Koodous Platform since the previous fetch. One analysis concerns one application. Each analysis is transformed into a feature vector describing the application. The vector is stored in local storage;
- Filling ratings (run once a day)—the process downloads the rating for each application downloaded in the previous process. The rating is collected at least 24 h after Koodous generates the analysis. The delay is needed for the community to evaluate the application. The length of the delay was experimentally estimated (see Section 3.2);
- Building a detection model (frequency depends on the strategy)—the process builds a new detection model based on the recent data. The resultant model is sent to BotSense Management System, which populates it further to edge devices.

To build an effective detection model the neural network intelligence (Neural Network Intelligence: https://nni.readthedocs.io/en/v2.0, accessed on 20 July 2022), the (NNI) framework is used. NNI is the AutoML toolkit for hyperparameter optimization and neural architecture search. It implements multiple tuners that guide hyperparameter search. Within this paper, we use a simple tree-structured Parzen estimator, one of the classic Bayesian optimization algorithms [33].

The k-fold approach was implemented to compare the quality of the models created with different sets of hyperparameter values. However, due to the time dependency of the malware characteristic, we split the data into learning and validation sets according to the application creation date—the model was built upon older samples and validated against newer ones. Thus, the obtained model parameters should be less sensitive for model aging, i.e., the phenomena of efficiency decreasing with time.

**Figure 6:**
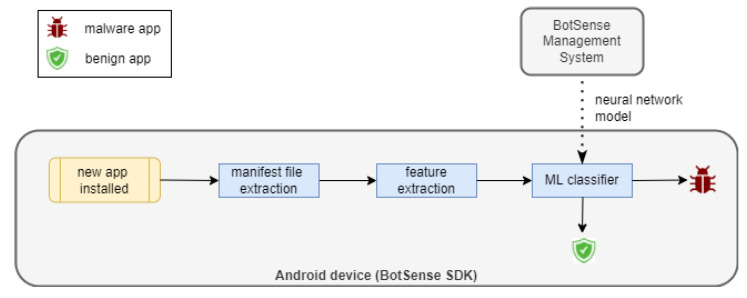
The process of assessing a newly installed application as malicious or benign.

The optimized neural network model is transferred to the edge device. Within the BotSense SDK, each newly installed application is detected and the manifest file of this application is extracted. Then, this file is used to compute the feature vector. Finally, the feature vector is used as the input of the neural network to assess the maliciousness of the application. The decision process is illustrated in Figure 6.

## 5. Experiments

The aim of the experiments was three-fold. First, to examine the quality of a malware predictor created using ML. The predictor is two-stage. First, the application is classified as malicious or benign. Then, the suspected sample level of malice is estimated. Secondly, we verify the automated model generation approach.

Additionally, the quality of data collected in Koodous was examined. Contrary to many previous studies on malware detection with ML, we focus on the problem of model aging. We propose and compare two model update strategies.

### 5.1. Dataset

Since with Koodous API, downloading newly arriving applications is much more efficient than the older data, the research was only carried out on newly added data. We started the data collection process on the 28 April 2022 and gathered 975.883 samples until the 21 June 2022.

**Figure 7:**
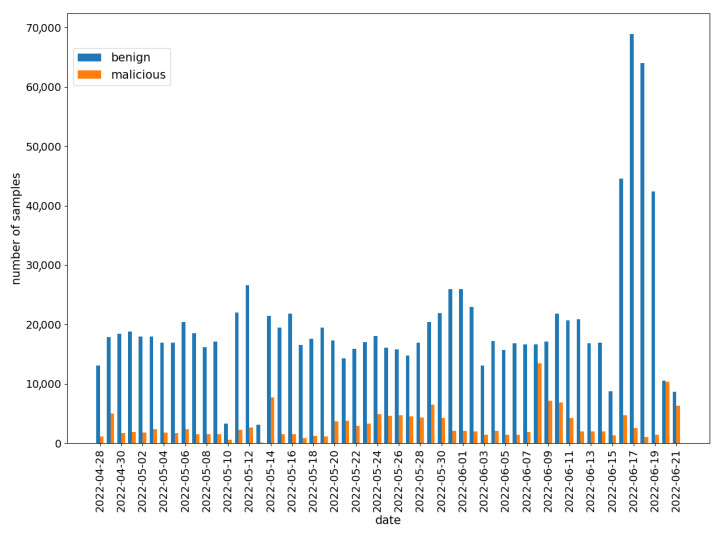
Labeled data distribution in Koodous from 28 April to 21 June.

Initially, the data were divided into benign and malicious. Samples with a feature rating of at least 0 were labeled as benign. Those with a negative rating were labeled as malicious. The daily distribution of samples was shown in Figure 7.

**Figure 8:**
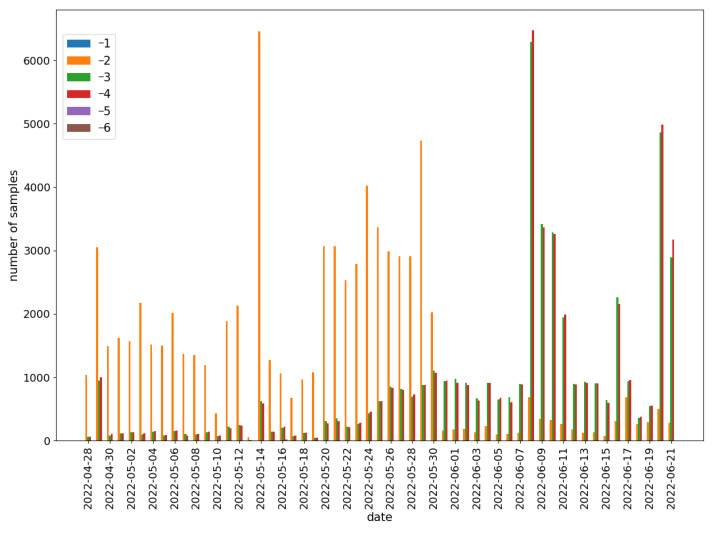
Labeled malicious data distribution in Koodous from the 28 April to the 21 June.

It can be observed that there are more benign samples than malicious ones. The number of benign samples is relatively stable (except at the end of June), while the number of malware varies significantly over time. A detailed distribution of the malware samples rating was presented in Figure 8.

The sudden change in the distribution of the rating values on the 31 May is noteworthy. Until the 30 May, the vast majority of malicious samples had a rating of −2. Then, the proportions were reversed in favor of the applications with lower rating values (rating∈{−3,−4}).

### 5.2. Model Ageing

When the model is built based on historical data, the problem of model aging occurs very often. The aging refers to the decaying performance of the model. This phenomenon is caused by changing data characteristics. In the case of mobile malware detection, the change of characteristics may be due to a new Android version release or a new malware family appearance. It is also a severe problem with detectors of other malware types.

The model should be constantly updated to solve the problem of model aging. In this paper, we examine two model update strategies:

- **INCREMENTAL**—the beginning of the training period is constant, and the end of this period is shifted by one day each day;
- **MOVING**—the beginning and the end of the training period are shifted by one day each day (the length of the training period is constant).

**Figure 9:**
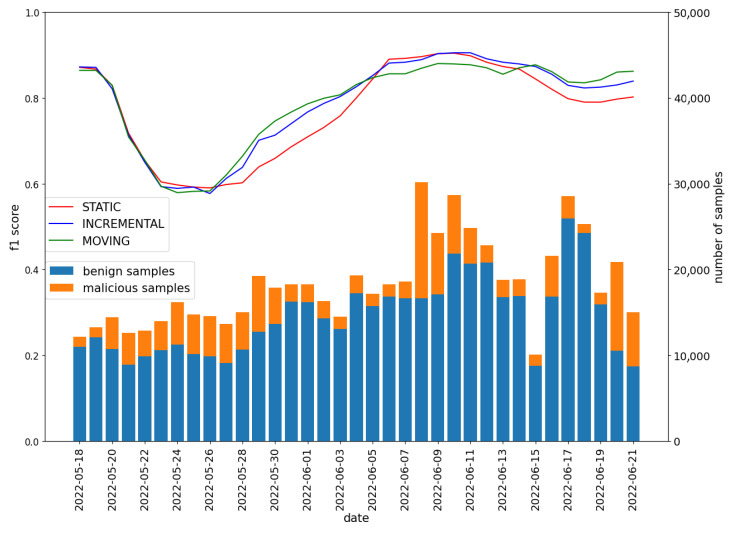
Comparisonof three updating model strategies (left-side axis) and the number of samples on each day of the experiment (right-side axis). Gradient boosting classifier was used to build classifiers.

We compare the results of both strategies with the **STATIC** approach—the training period is constant (no model update). The model was trained with the gradient boosting classifier. The initial length of the training period was two weeks in all cases. Each day, the testing set contained applications from the whole week following the training period. The obtained values of the f1 metric are presented in Figure 9. Note that the date on the axis X points to the last day of the test window (e.g., the 18 May indicates a test window that includes 12–18 May).

As can be observed in Figure 9, in the beginning, the choice of strategy does not affect the model quality. Then, the refreshing model lets the detector adapt to new data characteristics. However, interestingly, after the 6th of June, the model trained on old data (both **STATIC** and **INCREMENTAL** strategies) slightly outperformed the one with a moving training window. This may be due to the test data characteristics resembling those of the initial training data. Based on the results, it can be concluded that the **MOVING** strategy allows for the fastest adaptation to changing conditions.

On the other hand, keeping old data in the training set increases quality in most cases. In the future, some mixed approaches to the proposed updated strategy, i.e., the **INCREMENTAL** approach with lower weight of older samples, could be examined. However, in the short period of testing, as in this experiment, the **STATIC** approach allows for achieving good results.

An additional result of this experiment was discovering the most critical features for detecting malware. The five features that most affect the quality of the model for the **STATIC** strategy during training, along with their weight (weights of all 71 features add up to 1), are presented in Table 2. It can be noted that the most relevant features are the permission to read SMS by application and the number of URL addresses that the application uses.

### 5.3. On-Device Detector

As pointed out in the previous section, no mature tool allows deploying the GBC model on the device with the Android operating system. Thus, a more resource-exhaustive process of training neural networks is required. We applied the automatic approach described in Section 4.5 to obtain a high-quality model. The network was composed of three hidden layers (one optional) and the dropout layer (with this same value) after each hidden layer. The batch size and learning rate parameters were also adjusted. The hyperparameter search space definition is presented in Table 3. Zero neurons in the first hidden layer mean that this layer is skipped. The number of neurons in the third layer was limited to decreasing the risk of overfitting. The smallest value of the batch size was set to 32 to limit the memory and computation time used during training. Note that only the dropout parameter is chosen from a uniform distribution between 0 and 0.2, but all other parameters can be just one of the elements of the predefined list. The number of epochs was set to 8 in each trial.

The training set was composed of samples from 28 April to 9 May (12 days), while samples created the validation set from the next two days. The 500 trials were examined. The experiment was conducted on a machine with 64 GB of RAM, 8 CPUs and a Ubuntu 20.04.4 LTS operating system. The execution of all trials took approximately 400 min.

**Figure 10:**
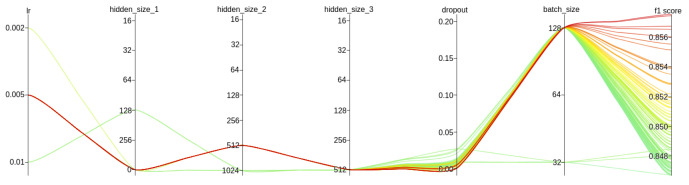
Top 20% trials of the hyperparameters search.

The result of the hyperparameters search is presented in Figure 10. For greater clarity, only the top 20% of trials (out of a total of 500 trials) are shown. The best model was created with the hyperparameter values outlined in Table 4. The selected metrics obtained on the validation set by this model are presented in the Table 5.

**Figure 11:**
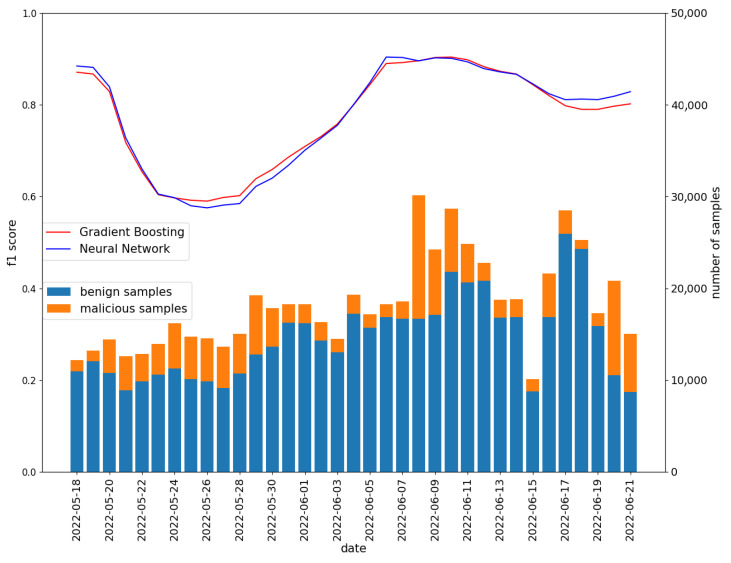
Comparing the results of chosen neural network and gradient boosting classifier.

Then, the Neural Network model was retrained on all samples from 28th April to 11th May with the chosen hyperparameters values and tested on data from 11th May to 21st June. Each day contained all data from the whole week that ended the selected day. The performance of the neural network was compared with the previously created model with gradient boosting classifier (the model trained with **STATIC** strategy described in Section 5.2). The results obtained by those models are presented in Figure 11. The neural network model behaves similarly to the gradient boosting classifier on all tested days. It can therefore be expected that conclusions regarding model aging are also valid for the neural network model. The selected metrics obtained on the test set (all days included) are presented in Table 6. The high precision of the model (0.9) means that, among the applications indicated by the network as malicious, the vast majority pose a threat to the user. Thanks to the low false positive rate (0.08), the user is rarely exposed to false alarms that could affect the user’s comfort.

This is a first work that evaluates an ML-based malware detector on a dataset labeled by the Koodous community. Thus, strict comparison with the state-of-the-art results is not possible. However, some general conclusions can be drawn:

- Similarly to work [29], we observed the degradation of the model accuracy with time—for most of the test datasets, the updated model performs better than the older one (see INCREMENTAL/MOVING vs. STATIC on Figure 9);
- The light neural network model achieves similar effectiveness as tree-based ML algorithms (see Figure 11). Tree-based approaches were commonly and successfully used in previous research. This is all the more important as the lightweight model was obtained as a result of the fully automated process;
- The effectiveness of the detection models varies over time—for some days, the model quality is similar to the one known from the previous works, but for others, it is significantly lower. The lower effectiveness is related to lower recall (increased number of undetected malware samples), which new malware families’ appearance might cause. However, it must be stressed that the model’s effectiveness strictly depends on the training and the evaluation datasets. As shown in this paper, the characteristics of the data in the Koodous Platform are highly variable in time.

### 5.4. Malignancy Degree Predictor

After a sample is classified as malicious, it is recommended to predict what degree of malignancy it may have so that the user risk assessment can be performed accurately. It is assumed that, the lower the rating, the more dangerous the sample is. To train a model to predict the degree of malignancy of a given application, supervised learning was performed, where the label of a given application is its rating. For this purpose, the regression suits better than classification, as the labels represent the degree of the malignancy (they can be ranked). In our research, the gradient boosting regressor was used.

The output of the regression model is a real number representing the predicted degree of malignancy. In the Koodous-based dataset, the model’s output is the expected rating of the malicious application, so the model’s output should be a real number from −6 to −1 or a little out of this range due to predictor error. The quality of the prediction will be assessed with the RMSE metric.

**Figure 12:**
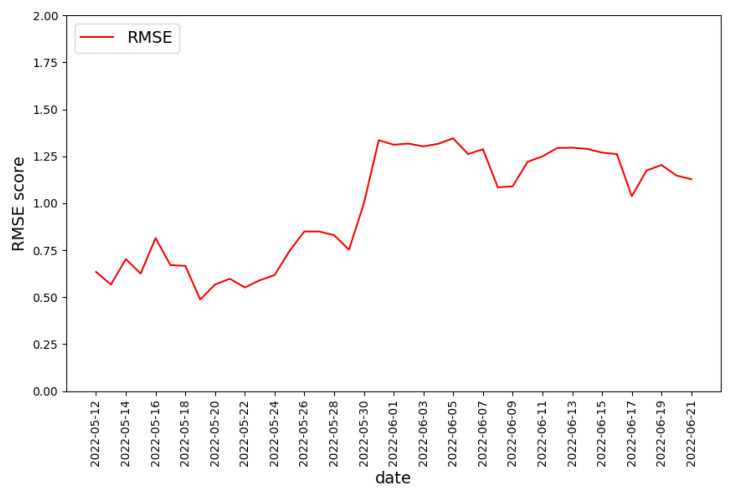
RMSE score for the predictor output predicting the degree of maliciousness of the application.

Again, the training set was composed of the malware samples from the first two weeks of the dataset. The predictor was tested against the rest of the data. The obtained results are presented in Figure 12. It can be observed that the predictor quality has drastically dropped since the 31 May.

If we compare the course of the RMSE value to the ranking distribution (Figure 8), it can be observed that the drop in RMSE value is associated with the change in ranking distribution, which happened on the 31 May. Reasons that could explain the significantly lower average ranking value are:

- New applications are more malicious, so they are easier to detect;
- New automatic malware detectors appeared on the Koodous platform;
- Existing malware detectors increased detectability.

Suppose new applications are more malicious but similar to the most malicious ones before 31 May. In that case, the RMSE value should not drop so significantly—the predictor estimated the rating of more malicious applications with high accuracy before the 31 May. Thus, it is more probable that the characteristic of malware detectors available in Koodous changed. If so, the application similar to the one in the training set might be treated as more malicious after the 31 May. If this statement is true, then it should be possible to calculate the bias value—the value indicating how many more negative votes obtain the malicious application after the 31 May. The bias value can be added to the result of the predictor to obtain a malignancy level more similar to the one in the Koodous platform. We obtained the bias value by optimizing the RMSE value of gradient boosting regressor for data collected on May 31 only. The resulting value is bias=−1.2.

**Figure 13:**
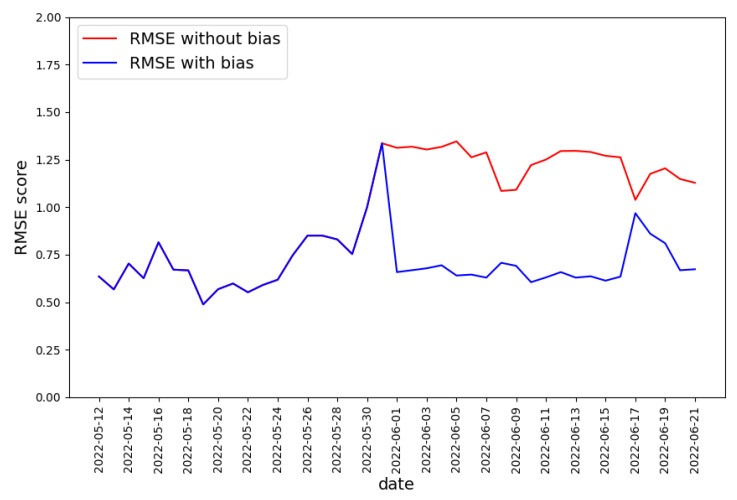
The RSME value obtained by the regression model in variants without and with applying bias value.

The bias value was added to the predictor output for all days after the 31st of May. The RSME value obtained by regression model in variants with and without applying bias value is presented in Figure 13. As expected, using the bias value increased the prediction quality to the level before 31 May, except for the data collected on 17 June. The results support the thesis that the data characteristics did not change, but their evaluation method has. Thus, the model should be frequently refreshed enough to keep the quality of the malignancy degree predictor similar to that on the Koodous platform.

## 6. Conclusions

Nowadays, malware detection on mobile devices is a great challenge. This paper proposes on-device malware detection based on Koodous-derived knowledge supported by an ML-based detector. The first detects malware that the Koodous research community detected using a blacklist. The latter is responsible for protecting users by detecting malware that has not yet been added to Koodous or has not been correctly labeled as malware by Koodous society. The detection tool was integrated within the BotSense Mobile system to increase the safety of users of e-banking and e-identity applications. The PyTorch framework was used to deploy the ML model on Android OS devices. In the future, the detection tool could be applied to detect attacks targeting other critical cyber–physical systems [34] based on Android devices (e.g., industrial control systems [35]).

The advantage of the Koodous platform is its ability to automatically download the applications with static analysis results. The number of applications appearing on the platform is also a significant advantage. Those features allow automating the process of generating new malware detection models. This is a promising solution as the characteristics of malicious applications change over time, causing model aging. Additionally, a Koodus-related label, namely rating attribute, allows for grading the maliciousness of the application. Predicting the degree of malignancy of the application may be of great importance in the comprehensive security assessment process carried out by the client.

In this work, we addressed the problem of model aging within the hyperparameters search process by modifying the k-fold approach—the validation set is composed of newer data than in the training set. We also proposed and compared two strategies for updating the malware detector model. We showed that frequent updates increase the detection quality in the short term. However, removing old data from the training set may decrease the model accuracy in the future. The computational cost of the retraining model should also be considered while choosing one of the updating strategies. The model aging was evident in the case of the malignancy degree predictor. However, according to the conducted research, the problem was rather related to the volatility of the Koodous platform than the model quality itself.

The preliminary studies show the satisfactory quality of the created models. In the case of neural network-based malware detection, the average $f_{1}$ score is 0.77, and for 60% of test datasets, the $f_{1}$ measure was higher than 0.8. The high precision of the model (0.9) is noteworthy. For malignancy level detection, the average MAE value was 0.49 (on average, the predicted ranking differed from the actual ranking by less than half of the vote), as long as the Koodous-related data labeling process was not changed.

The main limitation of this study is treating labels within Koodous as ground truth. The evaluation results indicate how well the created predictors forecast Koodous-based labels. The actual quality might be better (e.g., whether Koodous have samples that are FN and ML-based predictor discovers them as malware) or worse (if both Koodous and ML-based predictor mislabels the same applications). A preliminary study on the newest data presented in this paper shows a high variability of Koodous data characteristics even in a short period. In the future, we propose testing the models on third-party datasets to confirm the quality of the predictors. At the same time, the development of the machine learning libraries should be monitored for compatibility with the Android operating system. The emergence of new and mature tools will allow the use of more complex learning models, e.g., hybrid approaches [36] or ensemble models [27].

## Figures and Tables

**Table 1:** Overview of articles on applying machine learning methods for malware detection based on static analysis.

Paper	Dataset	ML Method	Evaluation Metrics	Results
Fauzia Idrees et al. [21]	Contagio Malware Dump MalGenome Google Store AppsApk Androidmob	Naive Bayes Decision tree Random forest MLP	accuracy	99.8%
Coronado-De-Alba et al. [23]	Drebin Google Store VirusTotal	Random forest	accuracy	97.5%
Alejandro Martin Garcia et al. [22]	OmniDroid	Random forest	accuracy	89.3%
Nektaria Potha et al. [27]	AndroZoo VirusShare Drebin	Logistic regression MLP SGD	AUC accuracy	99.5% 98.7%
Taheri et al. [28]	Drebin Contagio Mobile MalGenome	KNN	accuracy	90%–99%
Kouliaridis et al. [24]	AndroZoo	AdaBoost KNN Logistic regression Naive Bayes MLP SGD Random forest SVM	AUC accuracy	95.1% 91.7%
Arthur Fournieret al. [29]	theZoo contagio Google Store Drebin	Random forest Random committee Random tree IBK MLP Linear regression	accuracy	93.85%
Jin Li et al. [25]	Google Store	SVM Naive Bayes Decision tree	accuracy	95.63%
Durmus¸ Ozkan Sahin et al. [26]	APKPure AMD	MLP Naive Bayes Linear regression KNN Random forest SMO	$F_{1}$-score	98.5%

**Table 2:** The most relevant features with weights calculated by the gradient boosting classifier.

Feature Name	Feature Weight
READ_SMS	0.208
urls_count	0.195
RECEIVE_SMS	0.157
INTERNET	0.099
SYSTEM_ALERT_WINDOW	0.079

**Table 3:** Definition of the hyperparameter search space for building an on-device neural network model.

Parameter Name	Sampling Strategy	Values
Learning rate	choice	{0.002, 0.005, 0.01}
First hidden layer size	choice	{0, 16, 32, 64, 128, 256}
Second hidden layer size	choice	{16, 32, 64, 128, 256, 512, 1024}
Third hidden layer size	choice	{16, 32, 64, 128, 256, 512}
dropout	uniform	<0, 0.2>
batch size	choice	{32, 64, 128}

**Table 4:** Set of the hyperparameters for the Neural Network model with the best f1 score.

Parameter Name	Value
learning rate	0.005
First hidden layer	0
Second hidden layer	512
Third hidden layer	512
dropout	0
batch size	128

**Table 5:** Results obtained by the model trained on data from 28th April to 9th May with parameters presented in Table 4 and validated on data from 10th May to 11th May.

Accuracy	Precision	Recall	f1
0.86	0.86	0.85	0.86

**Table 6:** Results obtained by the model trained on data from 28 April to 11 May with parameters presented in Table 4 and tested on data from 11 May to 21 June.

Accuracy	Precision	Recall	f1
0.81	0.9	0.7	0.77

## Data Availability

Not applicable.

## Abbreviations

The following abbreviations are used in this manuscript:

SDK	Software Development Kit
SIEM	Security Information and Event Management
ML	Machine Learning
API	Application Programming Interface
APK	Android Package Kit
GBR	Gradient Boosting Regressor
GBC	Gradient Boosting Classifier
TP	True Positive
FP	False Positive
FN	False Negative
TN	True Negative
RMSE	Root Mean Square Error
MAE	Mean Absolute Error
NNI	Neural Network Intelligence
LTS	Long-Term Support

## Appendix A

Dangerous filters:

- ACTION_APPLICATION_RESTRICTIONS_CHANGED
- ACTION_CONFIGURATION_CHANGED
- ACTION_CREATE_SHORTCUT
- ACTION_INSTALL_PACKAGE
- ACTION_PACKAGE_DATA_CLEARED
- ACTION_PACKAGE_NEEDS_VERIFICATION

Dangerous permissions:

- ACCESS_CHECKIN_PROPERTIES
- ACCESS_COARSE_LOCATION
- ACCESS_FINE_LOCATION
- ACCESS_NOTIFICATIONS
- ACCESS_WIFI_STATE
- ADD_VOICEMAIL
- ANSWER_PHONE_CALLS
- BLUETOOTH_MAP
- BODY_SENSORS
- BROADCAST_WAP_PUSH
- CALL_LOG
- CALL_PHONE
- CAMERA
- CAPTURE_VIDEO_OUTPUT
- CHANGE_NETWORK_STATE
- CHANGE_WIFI_STATE
- DISABLE_KEY_GUARD
- GET_ACCOUNTS
- GET_TASKS
- INSTALL_PACKAGES
- INTERNET
- MANAGE_DOCUMENTS
- PERSISTENT_ACTIVITY
- PROCESS_OUTGOING_CALLS
- READ_CALENDAR
- READ_CALL_LOG
- READ_CONTACTS
- READ_EXTERNAL_STORAGE
- READ_HISTORY_BOOKMARKS
- READ_LOGS
- READ_PHONE_NUMBERS
- READ_PHONE_STATE
- READ_SMS
- READ_SYNC_SETTINGS
- RECEIVE_BOOT_COMPLETED
- RECEIVE_MMS
- RECEIVE_SMS
- RECEIVE_WAP_PUSH
- RECORD_AUDIO
- RECOVERY
- RESTART_PACKAGES
- SEND_SMS
- SET_ALWAYS_FINISH
- SET_WALLPAPER
- SYSTEM_ALERT_WINDOW
- USE_SIP
- WRITE_APN_SETTINGS
- WRITE_CALENDAR
- WRITE_CALL_LOG
- WRITE_CONTACTS
- WRITE_EXTERNAL_STORAGE
- WRITE_SETTINGS
